# Critical illness-related corticosteroid insufficiency in patients with severe acute biliary pancreatitis: a prospective cohort study

**DOI:** 10.1186/cc7978

**Published:** 2009-07-24

**Authors:** Yun-Shing Peng, Cheng-Shyong Wu, Yung-Chang Chen, Jau-Min Lien, Ya-Chung Tian, Ji-Tseng Fang, Chun Yang, Yun-Yi Chu, Chien-Fu Hung, Chih-Wei Yang, Pang-Chi Chen, Ming-Hung Tsai

**Affiliations:** 1 Division of Endocrinology, Chang Gung Memorial Hospital, 6, West Section, Chia-Pu Road, Chia-Yi 613, Taiwan; 2 Chang Gung Technology college, 2, West Section, Chia-Pu Road, Chia-Yi 613, Taiwan; 3 Division of Gastroenterology, Chang Gung Memorial Hospital, 6, West Section, Chia-Pu Road, Chia-Yi 613, Taiwan; 4 Chang Gung University, College of Medicine, 259, Wen-Hwa 1st Road, Kwei-Shan, Tao-Yuan 333, Taiwan; 5 Division of Critical Care Nephrology, Chang Gung Memorial Hospital, 199, Tung-Hwa North Road, Taipei 105, Taiwan; 6 Division of Gastroenterology, Chang Gung Memorial Hospital, 199, Tung-Hwa North Road, Taipei 105, Taiwan; 7 Department of Radiology, Chang Gung Memorial Hospital, 199, Tung-Hwa North Road, Taipei 105, Taiwan

## Abstract

**Introduction:**

Gallstones are the most common cause of acute pancreatitis worldwide. Patients with severe acute biliary pancreatitis (SABP) constitute a subgroup of severe acute pancreatitis (SAP) patients in whom systemic inflammation may be triggered and perpetuated by different mechanisms. The aim of this prospective investigation was to examine the adrenal response to corticotropin and the relationship between adrenal function and outcome in patients with SABP.

**Methods:**

Thirty-two patients with SABP were enrolled in this study. A short corticotropin (250 μg) stimulation test (SST) was performed within the first 24 hours of admission to the ICU. Critical illness related corticosteroid insufficiency (CIRCI) was defined as follows: baseline value less than 10 μg/dL, or cortisol response less than 9 μg/dL.

**Results:**

CIRCI occurred in 34.4% of patients. The patients with CIRCI were more severely ill as evidenced by higher APACHE II and SOFA scores and numbers of organ system dysfunction on the day of SST. The in-hospital mortality for the entire group was 21.9%. The CIRCI group had a higher hospital mortality rate compared to those with normal adrenal function (45.5% vs. 9.5%, *P *= 0.032). The hospital survivors had a higher cortisol response to corticotropin (17.4 (8.3–27.1) vs. 7.2 (1.7–12) μg/dL, *P *= 0.019). The cortisol response to corticotropin inversely correlated with SOFA score and the number of organ dysfunction on the day of SST. The rates of pancreatic necrosis and bacteremia were significantly higher in the CIRCI group (100% vs 42.9%, *P *= 0.002; 81.8% vs 23.8%, *P *= 0.003, respectively).

**Conclusions:**

CIRCI is common in patients with SABP. It is associated with bacteremia, multiple organ dysfunction and increased mortality.

## Introduction

Acute pancreatitis represents an acute inflammatory disorder with variable severity ranging from a mild, self-limited disease to a severe inflammatory cascade associated with multiple-organ dysfunction. Most mortality from acute pancreatitis is a consequence of multiple-organ dysfunction [1,2]. The precise mechanisms by which various etiological factors induce an acute attack are still unclear, but once the disease process is started, common inflammatory pathways are invoked. Initially, inflammatory reaction takes place within the pancreas, which can lead to systemic inflammatory response syndrome (SIRS); it is this systemic response that eventually contributes to multiple-organ dysfunction [3]. In fact, there is a bimodal distribution of mortality from acute pancreatitis. Approximately one-half of all mortality cases occurs early with a severe attack that results from the development of SIRS and subsequent multiple-organ dysfunction. Patients with severe acute pancreatitis (SAP) who die later in the clinical course often succumb to septic complications [1,2]. Despite improvements in critical care, early mortality remains a major contributory factor to overall mortality from acute pancreatitis, and continues to represent a clinical challenge [4,5]. It has been shown that early multiple-organ dysfunction syndrome (MODS) is not only responsible for the early mortality in patients with SAP, but it also identifies those patients most at risk of death from later septic complications [1]. The systemic effects of SAP share many similarities with those of other critical illness such as severe sepsis, liver failure, burns, and trauma [6-8]. They are all characterized by systemic inflammation, which potentially results in single-organ or multiple-organ dysfunction.

Critical illness is accompanied by the activation of the hypothalamic-pituitary-adrenal axis, which is highlighted by increased serum corticotropin and cortisol levels [9,10]. The activation of the hypothalamic-pituitary-adrenal axis is a crucial component of the host's adaptation to severe stress. Cortisol is essential for the normal function of the immune system and various cellular functions. Recently, the concept of critical illness-related corticosteroid insufficiency (CIRCI) has been put forward to describe a subnormal adrenal response to adrenocorticotropin in severe illness, in which the cortisol levels, although high in terms of absolute value, are inadequate to control the inflammatory situation [7-11]. The short corticotropin stimulation test (SST) is most commonly used to evaluate the appropriateness of the adrenal response in this setting [9,11]. Indeed, in patients with septic shock, a decreased response to the SST, namely an absolute increment of the serum cortisol level less than 9 μg/dL, is associated with an increased mortality [12-14]. Recently, accumulating evidence has suggested that CIRCI may also be involved in the pathogenesis of systemic inflammation in SAP [15-18].

Gallstones are recognized as the leading cause of acute pancreatitis worldwide [19]. In contrast to other etiological entities, the natural history of acute biliary pancreatitis is characterized by higher rates of bacteremia, cholangitis, pancreatic abscess, and infected necrosis; on the other hand, it is also marked by lower incidences of pseudocysts, splenic vein thrombosis, and chronic pancreatitis [19]. Recently, bacteremia has been identified as an independent factor associated with mortality in patients with acute pancreatitis [20]. In fact, bacteremia has also been shown to be an independent factor to predict CIRCI in patients with severe sepsis and septic shock [8,21]. Taken together, severe acute biliary pancreatitis (SABP) may constitute a subset in which the prevalence, mechanisms, and impacts of CIRCI may be different from those in other etiological entities. Indeed, patients with SABP may represent a subgroup of SAP patients in whom systemic inflammation is triggered and perpetuated by different mechanisms. Despite the growing interest in the association between adrenal dysfunction and SAP [22], adrenal responsiveness in SABP has, to the authors' knowledge, never been investigated explicitly. The aim of this investigation is to examine the adrenal response to corticotropin and the relation between adrenal function and outcome in patients with SABP.

## Materials and methods

### Patient information, data collection, and definitions

This study was conducted with approval from the institutional review board of Chang Gung Memorial Hospital, Taiwan, and in accordance with the Declaration of Helsinki of the World Medical Association. Written informed consent was obtained from the patients' relatives and next of kin This study was performed in the intensive care unit (ICU) of two university-affiliated hospitals between November 2004 and May 2006. The study enrolled 32 consecutive patients with SABP requiring intensive monitoring and/or treatment. The pancreatitis was considered to be of a biliary origin if gallstones were identified on ultrasonography or computed tomography (CT) scans and in the absence of other known etiological factors. Severe pancreatitis was defined according to the Atlanta criteria [23]. Patients were enrolled when one or more of the following were present: a Ranson score of three or higher, an Acute Physiology and Chronic Health Evaluation (APACHE) II score of eight or higher, or failure of one or more organs. Organ failure was defined as systolic blood pressure less than 90 mmHg, partial pressure of arterial oxygen (PaO_2_) less than 60 mmHg, or serum creatinine level greater than 2 mg/dL. Patients with a history of prior acute pancreatitis or corticosteroid treatment, and those who had received the steroidogenesis-inhibiting agent etomidate were excluded from this study. All ICU admissions were followed until discharge from the hospital or hospital mortality.

Vasopressor dependency was defined by a need for vasoactive substance(s) to maintain a systolic blood pressure greater than 90 mmHg despite volume expansion. Bacteremia was defined as the presence of viable bacteria in the blood [6], as evidenced by a positive blood culture up to three days before SST.

Organ function on the day of SST was evaluated using sequential organ failure assessment (SOFA) score [24]. Organ dysfunction was defined as previously described [18,24] and based on a score of two or more for any organ system in the SOFA score [24]. The hepatic scores were disregarded to preclude the confounding effects of obstructive jaundice induced by biliary stones. The cut-offs for dysfunctional organ systems were as follows: cardiovascular system, vasopressor dependency, namely dopamine at doses higher than 2 μg/kg/min or norepinephrine dobutamine at any dose; respiratory system, PaO_2_/fraction of inspired oxygen (FiO_2_) ratio less than 300; kidneys, serum creatinine level higher than 2 mg/dL; central nervous system, Glasgow coma score lower than 13; coagulation, platelet count lower than 100 × 10^9^/L.

Abdominal ultrasonography was performed for each case at presentation. Enhanced CT was performed when the disease was classified as severe. A CT-guided aspiration was performed and bacterial cultures were obtained when infected necrosis or abscess was suspected.

### Laboratory investigations

Blood cultures and appropriate cultures from the infection focus were obtained [25]. Prospectively collected information also included hematological and biochemical data, which are necessary to calculate various prognostic scores.

An SST was performed within the first 24 hours of admission to the ICU, with a median of three days (interquartile range (IQR) two to four) after the start of symptoms and a median of two days (IQR one to three) after admission to hospital. Synthetic adrenocorticotropic hormone 250 μg (Synacthen, Novartis Pharma AG, Basle, Switzerland) was given intravenously. Blood samples were obtained immediately before, and 30 and 60 minutes after injection. Cortisol levels were measured by a competitive immunoassay using direct chemiluminescent technology (Bayer Corporation, East Walpole, MA, USA). The peak cortisol level was defined as the highest cortisol level obtained following synacthen administration, whether at 30 or 60 minutes. The cortisol response was defined as the difference between the baseline and peak cortisol levels. The criteria for CIRCI were described previously [11] and are defined as follows: baseline value less than 10 μg/dL, or cortisol response less than 9 μg/dL.

### Statistical analysis

Results are expressed as median with IQR unless otherwise stated. Continuous variables were compared using the Mann-Whitney *U *test. Categorical data were tested using the chi-squared test. The correlation between the results of the SST and various prognostic scores was analyzed with linear regression using the Pearson method. All statistical tests were two-tailed, and the significance level was set at *P *≤ 0.05. Data were analyzed using SPSS 10.0 for Windows (SPSS Inc., Chicago, IL, USA).

## Results

### Subjects' characteristics

Thirty-two critically ill patients with SABP were enrolled in this investigation. The median patient age was 68 years. There were 12 men (37.5%) and 20 women (62.5%). Overall, the in-hospital mortality for the entire group was 21.9%.

Table 1 lists the patients' demographic data, clinical characteristics, and results of the SST for both survivors and non-survivors. The median number of organ dysfunctions on the day of SST was significantly higher among non-survivors (Table 1).

**Table 1:** Demographic data and clinical characteristics

	All Patients (n = 32)	Hospital Survivors (n = 25)	Hospital Non-survivors (n = 7)	*P *value
Age (years)	68 (55 to 79)	68 (55 to 81)	75 (55 to 80)	NS (0.293)
Gender (M/F)	12/20	9/16	3/4	NS (1.000)
BUN	15.5 (10.5 to 36.5)	14 (8.5 to 20.5)	40 (17 to 80)	< 0.001
Serum creatinine (mg/dl)	1.1 (0.6 to 1.9)	0.9 (0.6 to 1.4)	3.7 (2 to 4.2)	< 0.001
Bilirubin (mg/dl)	1.6 (0.8 to 3.2)	1.5 (0.7 to 2.6)	5.1 (1.2 to 11.5)	0.006
Albumin (g/l)	2.8 (2.4 to 3)	2.8 (2.4 to 3.1)	2.6 (2.3 to 2.8)	NS (0.090)
INR	1.2 (1.2 to 1.4)	1.2 (1.2 to 1.4)	1.3 (1.2 to 1.5)	NS (0.399)
MAP (mmHg)	79 (60 to 90)	81 (68 to 92)	60 (57 to 75)	0.041
SOFA score on the day of SST	5 (3 to 9)	4 (2.3 to 6.8)	12 (10 to 16)	0.001
APACHE II score on the day of SST	15 (10 to 20)	14 (9.5 to 18)	20 (15 to 36)	0.006
Ranson score	5 (3 to 6)	4 (3 to 5.5)	8 (5 to 9)	0.005
CIRCI (%)	11 (34.4)	6 (24)	5 (71.4)	0.032
Baseline cortisol (μg/dL)	21.6 (17.1 to 36)	20.7 (16.4 to 31.6)	28 (19.1 to 39.1)	NS (0.368)
Peak cortisol (μg/dL)	39.2 (32.1 to 46.8)	39.2 (32.5 to 47.1)	39.1 (29.7 to 46)	NS (0.316)
Cortisol increment (μg/dL)	14.5 (3.3 to 25.3)	17.4 (8.3 to 27.1)	7.2 (1.7 to 12)	0.019
Bacteremia (%)	14 (43.8)	10 (40)	4 (57.1)	NS (0.669)
Number of organ dysfunction on the day of SST	2 (1 to 3)	1 (1 to 2)	4 (3 to 5)	< 0.001
Respiratory dysfunction (%)	23 (71.9)	16 (69.6)	7 (100)	NS (0.149)
Coagulation dysfunction (%)	7 (21.9)	3 (12)	4 (57.1)	0.026
Cardiovascular dysfunction (%)	10 (31.3)	5 (20)	5 (71.4)	0.019
CNS dysfunction (%)	14 (43.8)	8 (32)	6 (85.7)	0.0287
Renal dysfunction (%)	12 (37.5)	5 (20)	7 (100)	< 0.001

APACHE = Acute Physiology and Chronic Health Evaluation; BUN = blood urea nitrogen; CIRCI = critical illness related corticosteroid insufficiency; CNS = central nervous system; F = female; INR = international normalized ratio; M = male; MAP = mean arterial pressure; NS = not significant; SOFA = sequential organ failure assessment; SST = short corticotropin stimulation test.

### Short corticotropin stimulation test

**Figure 1:**
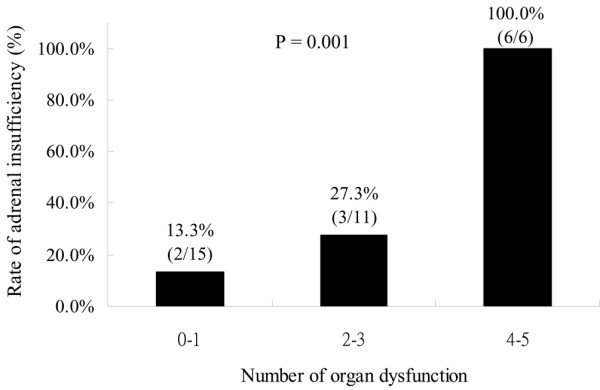
The rate of critical illness-related corticosteroid insufficiency increased progressively and significantly with the number of organ system dysfunctions.

As shown in Table 1, the response to corticotropin was significantly higher in those who survived, while the baseline and peak cortisol levels were not different between survivors and non-survivors. According to the aforementioned criteria, 11 (34.37%) patients had CIRCI. All 11 patients had a cortisol response less than 9 μg/dL. None of these 11 patients had a baseline level less than 10 μg/dL. The clinical characteristics and outcomes of patient subgroups stratified by adrenal functions are listed in Table 2. The ICU and hospital mortality rates of the patients with CIRCI were significantly higher than for those with normal adrenal function (45.5% vs 4.8%, and 45.5% vs 9.5%, respectively, *P *= 0.011 and 0.032, respectively). The incidence of CIRCI increased progressively and significantly with the number of organ system dysfunctions (chi-squared for trend, *P *= 0.001, Figure 1). The incremental response to corticotropin was negatively correlated with the SOFA score (R = -0.681; *P *< 0.001) and the number of organ system dysfunctions on the day of SST (R = -0.660; *P *< 0.001), while the baseline cortisol level was positively correlated with SOFA (R = 0.363, *P *= 0.045). However, there was no correlation between peak cortisol level and SOFA score on the day of SST.

**Table 2:** Demographic data and clinical characteristics grouped according to adrenal function

	All patients (n = 32)	Adrenal insufficiency (n = 11)	Normal adrenal function (n = 21)	*P *value
Age (years)	68 (55 to 80)	73 (58 to 80)	68 (55 to 81)	NS (0.685)
Gender (M/F)	12/20	4/7	8/13	NS (0.923)
ICU mortality (%)	6 (18.8)	5 (45.5)	1 (4.8)	0.011
Hospital mortality (%)	7 (21.9)	5 (45.5)	2 (9.5)	0.032
BUN (mg/dl)	15.5 (10.5 to 36.5)	23 (12 to 45)	15 (8.5 to 23.5)	NS (0.293)
Serum creatinine (mg/dl)	1.1 (0.6 to 1.9)	2.5 (1.1 to 3.7)	0.9 (0.6 to 1.3)	0.003
Bilirubin (mg/dl)	1.6 (0.8 to 3.2)	5.1 (1.9 to 9.6)	1.4 (0.7 to 2.0)	0.004
Albumin (g/l)	2.8 (2.4 to 3)	2.8 (2.4 to 2.8)	2.8 (2.2 to 3.1)	NS (0.839)
INR	1.2 (1.2 to 1.4)	1.3 (1.2 to 1.5)	1.2 (1.2 to 1.3)	NS (0.568)
MAP (mmHg)	79 (60 to 90)	58 (57 to 60)	84 (79 to 93)	< 0.001
SOFA score on the day of SST	5 (3 to 9)	11 (7 to 15)	4 (2 to 6)	< 0.001
APACHE II score on the day of SST	15 (10 to 20)	20 (10 to 27)	15 (11 to 18)	0.018
Ranson score	5 (3 to 6)	7 (5 to 9)	4 (3 to 5)	< 0.001.
Necrosis (%)	20 (62.5)	11 (100)	9 (42.9)	0.002
Bacteremia (%)	14 (43.8)	9 (81.8)	5 (23.8)	0.003
Number of organ dysfunction on the day of SST	2 (1 to 3)	4 (2.5 to 5)	1 (1 to 2)	< 0.001
Respiratory dysfunction (%)	23 (71.9)	9 (81.8)	14 (66.7)	NS (0.441)
Coagulation dysfunction (%)	7 (21.9)	7 (63.6)	0 (0)	< 0.001
CV dysfunction (%)	10 (31.3)	9 (81.8)	1 (4.8)	< 0.001
CNS dysfunction (%)	14 (43.8)	6 (54.5)	8 (38.1)	NS (0.465)
Renal dysfunction (%)	12 (37.5)	7 (63.6)	5 (23.8)	0.027

APACHE = Acute Physiology and Chronic Health Evaluation; BUN = blood urea nitrogen; CIRCI = critical illness related corticosteroid insufficiency; CNS = central nervous system; CV = cardiovascular; F = female; ICU = intensive care unit; INR = international normalized ratio; M = male; MAP = mean arterial pressure; NS = not significant; SOFA = sequential organ failure assessment; SST = short corticotropin stimulation test.

**Figure 2:**
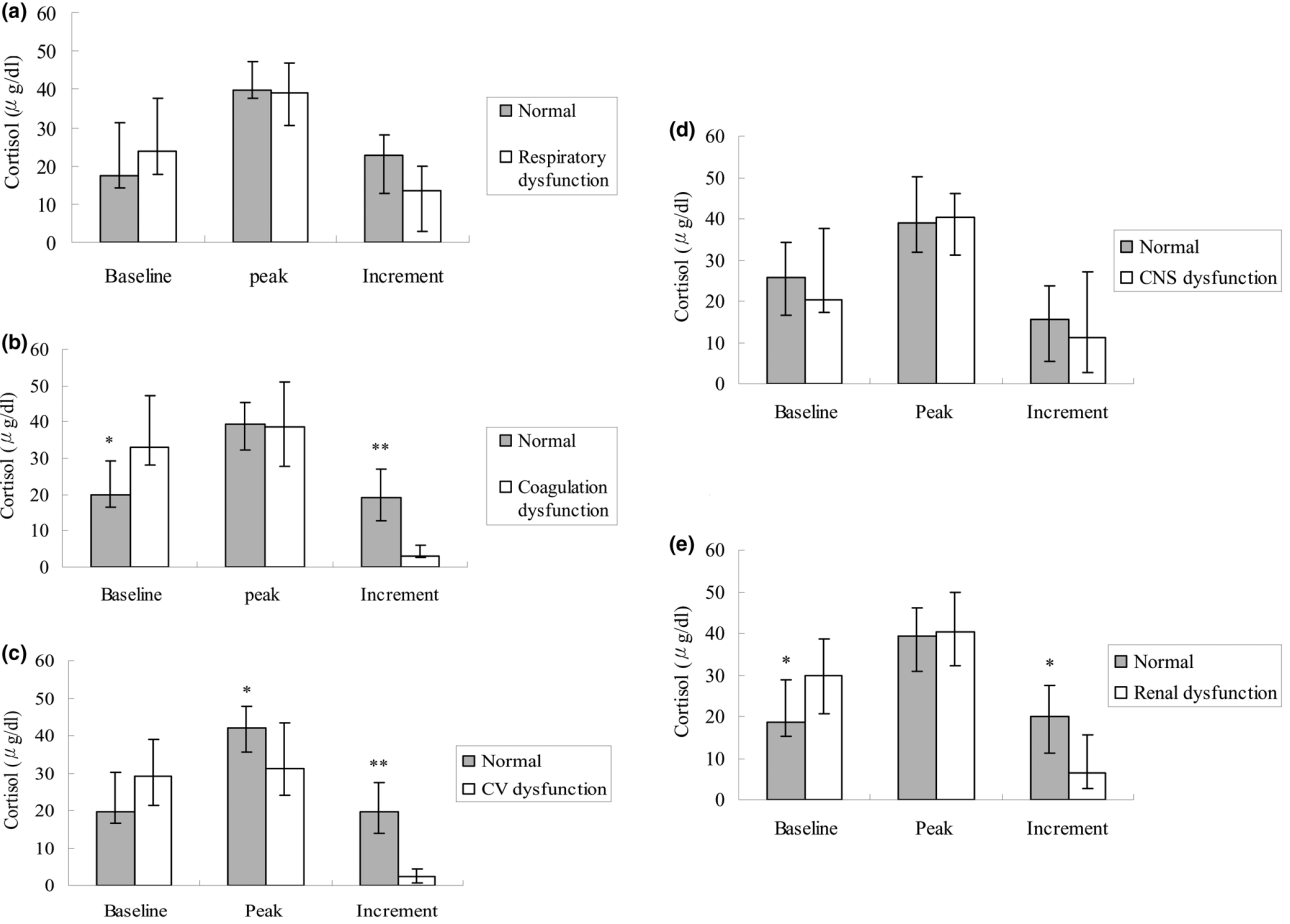
Results of the SSTs. **(a) **Results of the short corticotropin stimulation test (SST) in patients with respiratory dysfunction compared with those without respiratory dysfunction. **(b) **Results of the SST in patients with coagulation dysfunction compared with those without coagulation dysfunction. **(c) **Results of the SST in patients with cardiovascular (CV) dysfunction compared with those without CV dysfunction. **(d) **Results of the SST in patients with central nervous system (CNS) dysfunction compared with those without CNS dysfunction. **(e) **Results of the SST in patients with renal dysfunction compared with those without renal dysfunction. Results are expressed as median, with error bars representing the interquartile range. * *P *< 0.05; ** *P *< 0.01.

Patients with CIRCI had a more severe disease as evidenced by higher APACHE II and SOFA scores on the day of SST (Table 2). Additionally, the rate of pancreatic necrosis was significantly higher in the patients with CIRCI (Table 2). The results of SST when patients were grouped according to organ dysfunction were shown in Figure 2. On the day of the SST, 23 (71.8%) patients had respiratory dysfunction; 14 (43.7%) had central nervous system (CNS) dysfunction; 12 (37.5%) had renal dysfunction; 10 (31.3%) had cardiovascular dysfunction; 7 (21.9%) had coagulation dysfunction. The incremental increase of cortisol levels was significantly lower in patients with cardiovascular, coagulation, and renal dysfunctions, while the baseline cortisol levels were significantly higher in those patients with renal and coagulation dysfunctions (Figure 2). There was no difference in SST between those patients with respiratory, or CNS dysfunctions and those with satisfactory respiratory and CNS functions.

**Figure 3:**
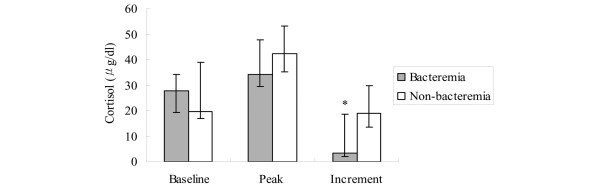
Results of the SST in patients with bacteremia compared with those without bacteremia. Results are expressed as median, with error bars representing the interquartile range. * *P *< 0.01. SST = short corticotropin stimulation test.

Microbiological information was available for all patients. Patients with bacteremia on the day of SST had a higher incidence of CIRCI compared with non-bacteremic patients (64.3% vs 11.1%, *P *= 0.003). The incremental increase of cortisol levels was significantly lower in patients with bacteremia (Figure 3).

## Discussion

This study shows that impaired adrenal function, as evidenced by the SST, is common in patients with SABP. CIRCI is associated with bacteremia, as well as increased rates of pancreatic necrosis, organ dysfunction, and mortality.

The evolution of organ dysfunction in patients with SAP has been described by Buter and colleagues [1]. Despite the bimodal distribution of mortality from SAP, the common cause of death is MODS [1]. Early MODS not only contributes to mortality in the early course of SAP but also represents the most significant non-fatal complication of SAP, causing major morbidity, and a strain on medical expenditure [26,27]. Consistent with previous investigations of patients with SAP [1,2], respiratory dysfunction is the common organ system dysfunction in SABP. However, hospital mortality in the present study was characterized by MODS in which respiratory dysfunction was accompanied by dysfunction of other organ systems. MODS occurring in the early stage of SAP share many similarities with severe sepsis and septic shock. The profiles of inflammatory mediators in SAP are similar to those in severe sepsis, suggesting that there may be common mechanisms behind uncontrolled inflammation and organ dysfunctions in both conditions [28]. The pathophysiology of MODS in SAP appears to be related to the systemic activation of various effector cells and inflammatory mediators that can act on remote organs [28]. Despite advances in the understanding of the pathophysiology of MODS in SAP, the outcomes of SAP remain unsatisfactory. In fact, attempts to ameliorate the SIRS using platelet activating factors failed to modify the course of MODS in SAP, suggesting that our knowledge of MODS in SAP is incomplete [29]. Recently, CIRCI has been recognized as an important phenomenon in the pathophysiological cascade of severe sepsis and septic shock [9,11]. It has also been shown that impaired adrenal response is associated with MODS and poor prognosis in patients with severe sepsis [30,31]. In our study, there was a negative correlation between cortisol increment and the number of organ system dysfunctions on the day of SST, suggesting that adrenal dysfunction is also related to MODS and poor prognosis in the setting of SABP. In this regard, accumulating lines of evidence indicate that CIRCI may contribute to the amplified systemic inflammatory response and modify the severity and pathological course of acute pancreatitis [15,18,32,33]. In fact, Abe and colleagues have demonstrated in experimental models of acute pancreatitis that inflammation is more severe and mortality is increased in adrenalectomized rats, suggesting that endogenous glucocorticoid may play an important role in mitigating the progress of inflammation [32]. Endogenous glucocorticoids may also play an important role in protecting acinar cells by decreasing their sensitivity to apoptosis during acute pancreatitis, thus suggesting that an inadequate glucocorticoid response in SAP can facilitate pancreatic necrosis [33].

Consistent with the previous observation of non-selected patients with SAP [18], the non-survivors among our patients with SABP had significantly lower cortisol increments to adrenocorticotropic hormone stimulation, suggesting an impaired anti-inflammatory response in those patients who succumbed. In the present study, all the patients with CIRCI developed pancreatic necrosis, although the causal relation between CIRCI and the formation of necrosis has not yet been definitively determined.

Cardiovascular dysfunction is a frequent complication of SAP. It also represents a risk for mortality [2]. In the present study, we showed that cardiovascular dysfunction was associated with CIRCI and hospital mortality in patients with SABP. Like vascular hyporeactivity to vasopressor in sepsis, patients with occult adrenal dysfunction have an impaired responsiveness to norepinephrine [34]. Indeed, steroid replacement may reverse the blunt response to vasopressor and improve the outcomes of septic patients with adrenal dysfunction [35,36]. These immunologic and hemodynamic effects of hydrocortisone in severe sepsis may result from the inhibition of cytokines and nitric oxide [37], which also mediate systemic inflammation and hemodynamic impairment in SAP [3,28]. In fact, steroid administration can also reduce vasopressor dependency in SAP with shock, as was found in a retrospective case-controlled study [16]. Considering the recent interest in the anti-inflammatory treatment of SAP, a subset of patients with SAP may benefit from glucocorticoids, owing to their anti-inflammatory effects and benefits in SAP with CIRCI. A prospective randomized study is needed to clarify the risks and benefits of glucocorticoid treatment in patients with SAP.

We observed that bacteremia is associated with CIRCI in patients with SABP. Patients with SAP are not an entirely homogenous group in terms of etiological factors. It has been shown that SABP is associated with higher rates of cholangitis and bacteremia [19,38,39]. In fact, bacteremia has been suggested to be a prognostic marker to predict infected necrosis and poor outcome in acute pancreatitis [20]. On the other hand, bacteremia has been shown to be an independent factor that predicts CIRCI in patients with severe sepsis and septic shock [8,21]. The presence of viable bacteria in the blood may reflect a higher bacterial load in a more immunocompromised host. In the current investigation, the cortisol increment was significantly lower in the bacteremic group (Figure 3), implying altered adrenal synthesis and responsiveness in this specific subset of patients. Considering the increased bacteremic events associated with SABP and the prognostic significance they may carry, further investigations into the pathophysiology of impaired adrenal function may help in improving the treatment strategy in this clinical setting.

## Conclusions

CIRCI is common among patients with SABP. CIRCI is associated with bacteremia, MODS, and increased mortality, and it occurs more frequently in patients with more severe disease. Whether glucocorticoid supplements in this subset of patients can mitigate multiple-organ dysfunction and improve outcomes remains to be clarified.

## Key messages

• CIRCI is a common phenomenon in patients with SABP.

• CIRCI is associated with bacteremia, multiple-organ dysfunction, and a poor outcome in patients with SABP.

• Clinicians should consider adrenal function tests in SABP patients with bacteremia and multiple-organ dysfunction.

• A prospective randomized study is needed to clarify the risks and benefits of glucocorticoid treatment in patients with SABP.

## Abbreviations

APACHE: Acute Physiology and Chronic Health Evaluation; CIRCI: critical illness-related corticosteroid insufficiency; CNS: central nervous system; CT: computed tomography; FiO_2_: fraction of inspired oxygen; ICU: intensive care unit; IQR: interquartile range; MODS: multiple-organ dysfunction syndrome; PaO_2_: partial pressure of arterial oxygen; SABP: severe acute biliary pancreatitis; SAP: severe acute pancreatitis; SIRS: systemic inflammatory response syndrome; SOFA: sequential organ failure assessment; SST: short corticotropin stimulation test.

## Competing interests

The authors declare that they have no competing interests.

## Authors' contributions

MHT conceived the study. CSW participated in its design and coordination. YSP participated in its design and coordination, and drafted the manuscript. All authors approved the manuscript after critical reading.
